# **Timely identification of atypical acute aortic dissection in the emergency department:a study from a tertiary hospital**

**DOI:** 10.3906/sag-1808-96

**Published:** 2019-10-24

**Authors:** You-Jin JIANG, Zheng-Fang ZHANG, Zhi-Ming GU, Heng-Di ZOU, Wen-Hui FAN, Xiao-Jun CHEN, Hong-You WANG

**Affiliations:** 1 Department of Emergency, Maanshan People’s Hospital, Maanshan, Anhui China

**Keywords:** Acute aortic dissection, atypical symptoms, assessment, diagnosis, D-dimer, computed tomography, computed tomographic angiography

## Abstract

**Background/aim:**

Acute aortic dissection (AAD) is a rare but fatal disease if left untreated. Symptoms are often similar to common conditions; therefore, the diagnostic strategy is important. We aimed to identify the atypical symptoms in a timely manner without putting patients at greater risk for undetected AAD.

**Materials and methods:**

We conducted a retrospective observational study of 59 AAD patients with both atypical and typical symptoms from January 2012 to December 2016. Patients with atypical symptoms continuing more than 30 min underwent a D-dimer test and computed tomography (CT) or computed tomographic angiography (CTA).

**Results:**

Of the 59 AAD patients, 22 were atypical. In the atypical group, the median delay time in our hospital was 3.1 h; average delay time after July 2015 was shorter than average delay time before June 2015 (16.59 ± 24.70 vs. 1.90 ± 0.57 h, P = 0.076).

**Conclusions:**

For patients in the emergency department who are suspected of having AAD, incorporating atypical symptoms with high levels of D-dimer into a triage strategy could improve the efficiency of clinical decision making. Furthermore, essential education directed towards the recognition of the atypical symptoms of AAD for front-line physicians may aid in a timely diagnosis, as compared with the usual assessments in the emergency department.

## 1. Introduction

Acute aortic dissection (AAD) is a rare but often catastrophic disease. Prompt and accurate diagnosis is crucial for survival. The incidence of aortic dissection has been steadily increasing, from 29 to 150 cases per 1 million of the population per year during a more recent period [1–3]. This increase may be attributed to the improved diagnostic equipment and case ascertainment [1–6]; however, a correct diagnosis of AAD is made in only 15% to 43% of patients initially thought to have the disease. Irrespective of the underlying mechanism, there is an obvious increase in prevalence [1–3,7]. Previous hospital-based studies from specialized centers or studies from retrospective registry data [1–3,8,9] indicate that a delay of 4.3 to more than 24 h occurs between presentation and diagnosis of AAD [9]. According to the records of untreated patients, the associated mortality is 1% per h immediately after the onset of symptoms [1,2,7]; however, previous studies have not detailed sufficient information during this interval or indicated how to shorten the delay between patient arrival and identification of AAD. The present study provides an in-depth discussion on how to identify atypical patients promptly and suggests diagnostic strategies to decrease delays in the diagnosis of AAD.

AAD is classified based on the anatomic distribution of the dissection, time from symptom onset, and presence of complications. However, the diagnosis of AAD is particularly challenging due to a combination of highly heterogeneous clinical presentation symptoms. Clinical guidelines suggest that AAD should be considered in all patients presenting with chest pain, back pain, abdominal pain, and syncope, or symptoms consistent with perfusion deficit, but these symptoms account for large proportions of emergency medical visits worldwide [1,7]. Validated diagnostic strategies are therefore needed to assist clinical evaluation and pay more attention to atypical patients without typical symptoms.

## 2. Materials and methods

### 2.1. Selection of patients

This study was a retrospective observational study of prospectively collected data to evaluate the efficacy and safety of the diagnostic strategy for atypical AAD from January 1, 2012, to December 31, 2016. Collected data included records of dates and times of symptom onset, clinical presentations, medical histories, physical findings, laboratory examinations, imaging use and results, emergency management, and outcomes. We incorporated patients’ actual descriptions into the following definitions of atypical symptoms—theseinclude new orchange in intensity or frequency and intermittent—they are: (1) light back or abdominal pain, (2) feeling of impending death, (3) unusual fatigue, (4) dyspnea, etc. Atypical symptoms are noncontinuous severe chest, back, and abdominal pain, palpitations, etc.; in other words, persistent acute symptoms are not the atypical ones. The geographic sector of this study includes the metropolitan area of Maanshan and its affiliated county, Dangtu, Anhui, China, which is comprised of a stable population of approximately 1,200,000 people in the time frame of our study period. All cases of AAD confirmed by computed tomography (CT) or computed tomographic angiography (CTA) were either operated on or treated with endovascular stent-graft placement or drugs in our hospital and at Nanjing Drum Tower Hospital, the Affiliated Hospital of the Medical School of Nanjing University, Nanjing, Jiangsu, China. Patients older than 18 years were selected, resulting in inclusion of 71 patients during the study period; 59 patients were diagnosed in the emergency department and were enrolled in the study. The remaining 12 patients were diagnosed during the duration of their hospital stay and were not included in the study because they did not visit our emergency department.

All 59 individuals diagnosed with AAD in our emergency department underwent cross-sectional imaging of the chest and/or abdomen using spiral CT/CTA to confirm an AAD diagnosis. If a differential diagnosis was needed, further examinations included a D-dimer test, electrocardiography (ECG), and cardiac enzyme tests. AAD was defined as the separation of the aortic wall layers with resulting true and false lumens or intramural hematoma.

This study was conducted in accordance with the declaration of Helsinki. This study was conducted with approval from the Ethics Committee of Maanshan People’s Hospital. Written informed consent was obtained from all participants (2017–009).

### 2.2. Laboratory examinations and triage course

**Figure 1:**
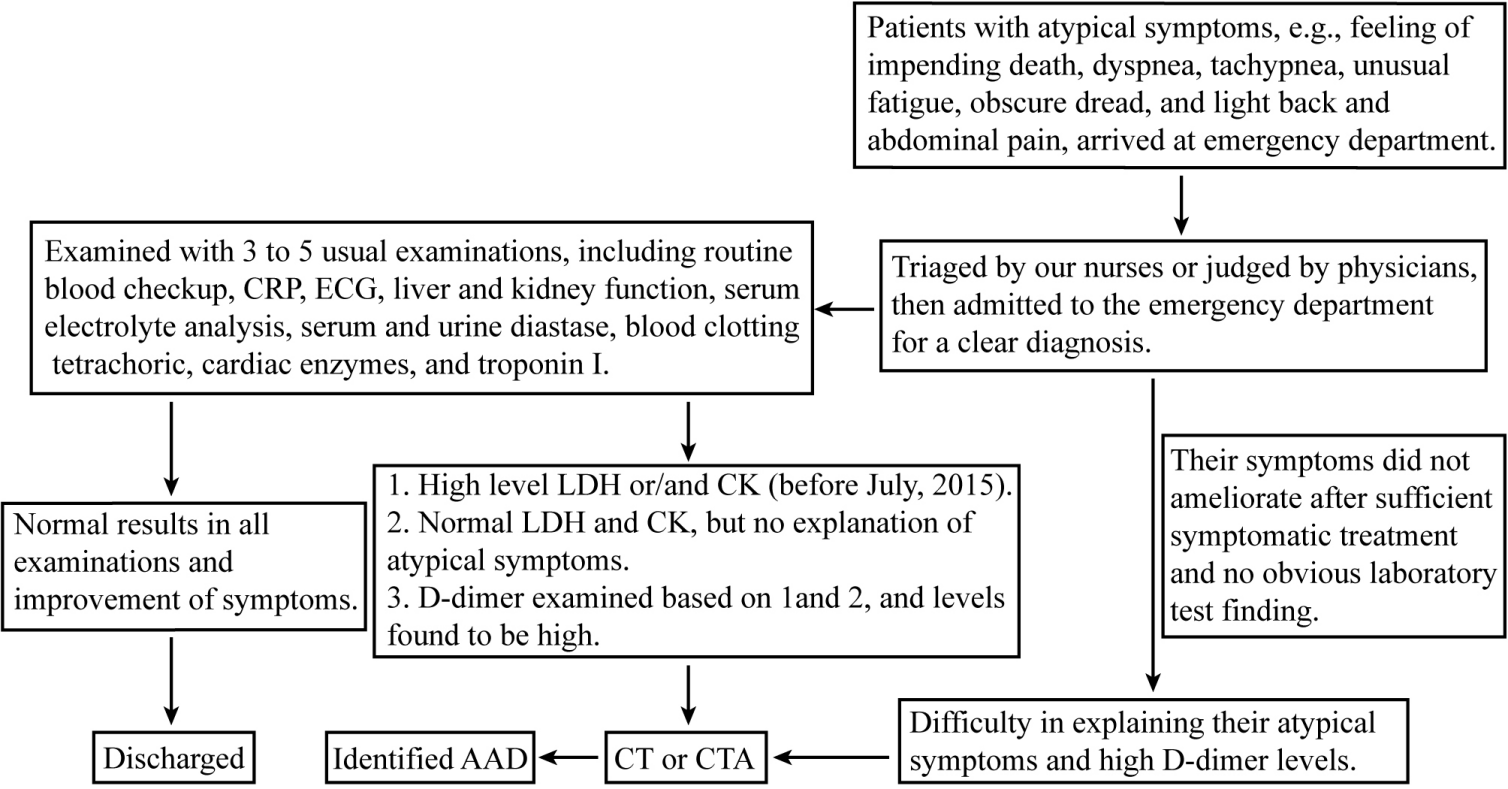
Diagram of AAD patients with atypical symptoms.

When patients with atypical symptoms arrived in our emergency department, they underwent three to five routine emergency laboratory examinations as follows: routine blood checkup, C-reactive protein (CRP), ECG, liver and kidney function, serum electrolyte analysis, serum and urine diastase, blood clotting tetrachoric, cardiac enzymes, and troponin I. When we found high levels of lactate dehydrogenase (LDH) and/or creatine kinase (CK) (before July, 2015) and the patient had difficulty in explaining their atypical symptoms, we administered the D-dimer test; a CT or CTA was indicated according to the result of the D-dimer test [10–16] because rapid diagnosis of AAD is possible when CT or echocardiography are part of the diagnostic testing [4,6,17–19]. Thus, we performed imaging for patients with suspected AAD using a helical CT scanner of 64 or 16 to identify their diagnosis as soon as possible. A diagram on how we identified AAD patients with atypical symptoms is shown in Figure 1.

**Table 1:** Baseline characteristics and presenting symptoms of patients with acute aortic dissection.

	Atypical group (%)n = 22	Typical group (%)n = 37	P-value
Age (years)	56.73 ± 13.85	64.19 ± 11.69	0.031
Male	16 (72.73)	24 (64.86)	0.532
Hypertension	7 (31.82)	31 (83.78)	<0.001
Presenting symptoms			
Severe chest pain	0	25 (67.57)	<0.001
Severe back pain	0	9 (24.32)	0.012
Severe abdominal pain	0	3 (8.11)	0.170
Palpitations	17 (77.27)	21 (56.76)	0.111
Tachypnea	15 (68.18)	29 (78.38)	0.384
Obscure dread	13 (59.09)	3 (8.11)	<0.001
Unusual fatigue	12 (54.55)	0	<0.001
Feeling of impending death	10 (45.45)	0	<0.001
Dyspnea	9 (40.91)	3 (8.11)	0.002
Light back or abdominal pain	7 (31.82)	0	<0.001
AAD, Stanford type B	15 (68.18)	22 (59.46)	0.774
Time from admission to a definitediagnosis delay time (median [min])	186	31	0.006
≤1 h	1 (4.55)	35 (94.60)	0.001
1–2 h	7 (31.82)	1 (2.7)	0.002
2–4 h	8 (36.36)	1 (2.7)	<0.001
>4 h	6 (27.27)	0	<0.001
	Time from admission to a definite diagnosis in atypical group (h)From January 2012 to June 2015 From July 2015 to December 2016		
	16.59 ± 24.70	1.90 ± 0.57	0.076

Note: Statistical difference between the two groups exists for some symptoms including severe chest and back pain, obscure dread, unusual fatigue, feeling of impending death, dyspnea, light back or abdominal pain and delay time, while others are not statistically significant.

We found that CK and LDH had the same significant value in AAD diagnostic course between atypical and typical group (CK, P = 0.877; LDH, P = 0.615), but the delay time was shorter after July 2015 (before July 2015, 16.59 ± 24.70 h, after July 2015, 1.90 ± 0.57 h, P = 0.076) because we provided our front-line physician education regularly and discussed every atypical AAD in our department before July 2015.

### 2.3. Assessment in the emergency department

According to the first evaluation by triage nurses, patients arrive at our emergency department and are assigned to different departments, such as internal medicine, emergency surgery, or the intensive-care room of the emergency department (IRED). Based on the physician’s judgment, patients in our IRED can be immediately hospitalized, referred, and consulted by related specialists and even several directors; repeated evaluations and new diagnostic strategy are carried out if a patient’s illness is complicated and a diagnosis could not be identified in time.

Within our department, patients who are considered to have a high or moderate risk must have a definitive diagnosis. Patients in whom it is difficult to identify a diagnosis during the night shift must stay in the emergency department and await a decision the next morning. All admissions are referred or approved by a related specialist, which differs from the emergency department principles of the United States of America and other western countries [17]. We study every atypical AAD patient and discuss their diagnostic course with our front-line physicians.

### 2.4. Clinical management

Intravenous antihypertensive treatment should be started urgently in all patients with hypertension, except in those with hypotension, as soon as AAD is suspected. The aims of medical therapy are to ease the systemic arterial pressure to as low a level as possible (around 100 mmHg) [20]. A combination of ß-blocker and vasodilator (i.e., sodium nitroprusside) is the standard medical therapy used in patients with suspected AAD [7,20]. Due to limited conditions, we prescribe 50–100 mg metoprolol orally, if without contraindications, to achieve a target heart rate of 60 beats per min, alongside intravenous sodium nitroprusside. Intravenous opiate analgesia is one of the most significant agents for AAD patients; intravenous morphine at 3 to 5 mg every 30 min will not only relieve severe chest pain, but also augment the effects of heart rate control and vasodilator agents.

### 2.5. Statistical analysis

We report data for the normal and skewed distributions as mean ± standard deviation (SD) or as median (IQR) and others as frequency (percentages). Normally distributed variables compared between two groups use Student’s t-test and others use the Mann–Whitney U-test. Categorical variables were compared by the Pearson chi-square test or Fisher’s exact test. P-values of less than 0.05 were considered to indicate statistical significance. All analyses were carried out using SPSS (version 18.0; IBM SPSS Statistical, NY, USA).

## 3. Results

### 3.1. Groups of patients

We found 59 AAD patients during our study period according to their diagnostic course; 22 atypical and 37 typical AAD patients were enrolled in the study. Ten atypical patients from July 2015 were diagnosed according to the presence of atypical symptoms and an increased D-dimer, without examination of LDH and CK levels. The average age was 56.73 ± 13.85 years in the atypical group and 64.19 ± 11.69 years in the typical group (t = 2.212, P = 0.03). The sex distribution was 16 males and 6 females in the atypical group, and 24 males and 13 females in the typical group (χ2 = 0.391, P = 0.53). There were 15 (68.18%) type B and 7 (31.82%) type A patients in the atypical group and 24 (64.86%) type B and 13 (35.14%) type A patients in the typical group (χ2 = 0.068, P = 0.79).

### 3.2. Clinical manifestations

Twenty-two (37.29%) of the atypical AAD patients did not experience severe chest pain during the study period. Sudden prolonged chest pain was the initial symptom in 25 (42.37%) of the 59 patients, often with a sharp, searing pain lasting no more than 30 min, followed by a painless or tolerable pain period. Abdominal pain was the first symptom in 9 (15.25%) of the 59 patients, which was often associated with severe cramps and shared the same time interval as the chest pain; however, these patients were younger and had few physical finds. Back pain was the first symptom in 3 (5.09%) of the 59 patients. AAD without pain occurred in 15 (25.42%) of the 59 patients with normal vital signs; however, they had the following atypical symptoms of AAD: palpitations, feeling of impending death, tachypnea, dyspnea, unusual fatigue, obscure dread, and light back and abdominal pain.The atypical symptoms and other relative characteristics in the atypical AAD patients are shown in Table 1. Even with sufficient symptomatic treatment, their symptoms did not improve or become worse.

### 3.3. Laboratory examinations

Of the 12 atypical AAD patients from January 2012 to June 2015, all had undergone urgent examination of LDH (range 313–618 IU/L) and CK (range 55–170 IU/L) levels. Three abnormal LDH and 3 abnormal CK results were found in 5 patients; the range of LDH was 202–850 IU/L and of CK 40–448 IU/L. Nine of the 12 atypical AAD patients underwent the D-dimer test (range 0–550 µg/L), 8 of which had abnormal results with a range of 390–4380 µg/L. Of the 31 typical AAD patients from January 2012 to June 2015, 14 underwent an examination of LDH and CK. Abnormal LDH and CK results were found in 7 and 4 of 7 patients, respectively; LDH ranged from 462–870 IU/L and CK from 42–283 IU/L. Seventeen typical AAD patients underwent a D-dimer test; 13 patients had abnormal results, ranging from 160–71,200 µg/L. The Pearson chi-square test was used to compare the atypical and typical groups (LDH, P = 0.615; CK, P = 0.877; D-dimer, P = 0.63).

### 3.4. Delay time in the emergency department

**Figure 2:**
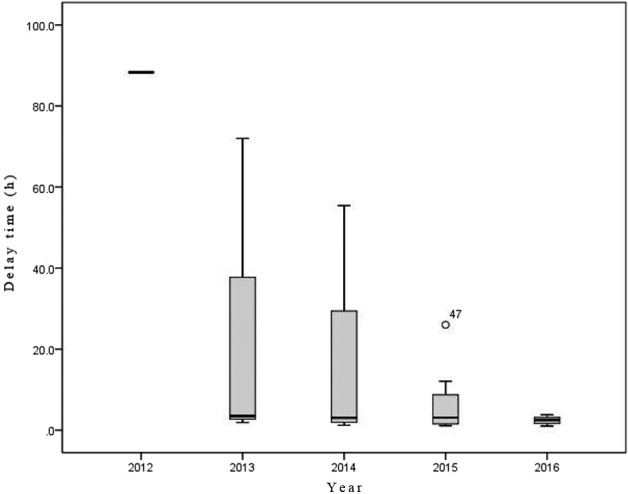
Delay time in atypical AAD patients according to quantity, with median (box), and IQR (bold line).

The median delay time of 22 atypical patients in our emergency department was 3.1 h (minimum: 1.0 h; maximum: 88.3 h; IQR: 5.4 h). The mean delay time of the atypical AAD patients diagnosed in the emergency setting was 88.3 h in 2012, 25.80 ± 40.02 h in 2013, 15.68 ± 26.50 h in 2014, 7.26 ± 9.12 h in 2015, and 2.41 ± 1.02 h in 2016. The statistical results are presented in Figure 2 and Table 2.

**Table 2:** The findings of age and sex of 59 AAD patients by year.

	2012 (%)	2013 (%)	2014 (%)	2015 (%)	2016 (%)
No.	7	9	13	18	12
Male	6 (85.71)	7 (77.78)	9 (69.23)	11 (61.11)	8 (66.67)
Age in atypical group※		63.67 ± 6.35	65.25 ± 13.70	55.29 ± 14.82	52.14 ± 14.78
No. of atypical AAD	1 (14.29)	3 (33.33)	4 (30.77)	7 (38.89)	7 (58.33)
Delay time in atypical group§		25.80 ± 40.02	15.68 ± 26.50	7.26 ± 9.12	2.41 ± 1.02
CT in atypical group			2 (50)	2 (28.57)	3 (42.86)

Note: ※P = 0.532, §P = 0.583. Delay time in atypical group is not statistically significant during five years but shows an obvious decline tendency.

### 3.5. Imaging and testing

**Figure 3:**
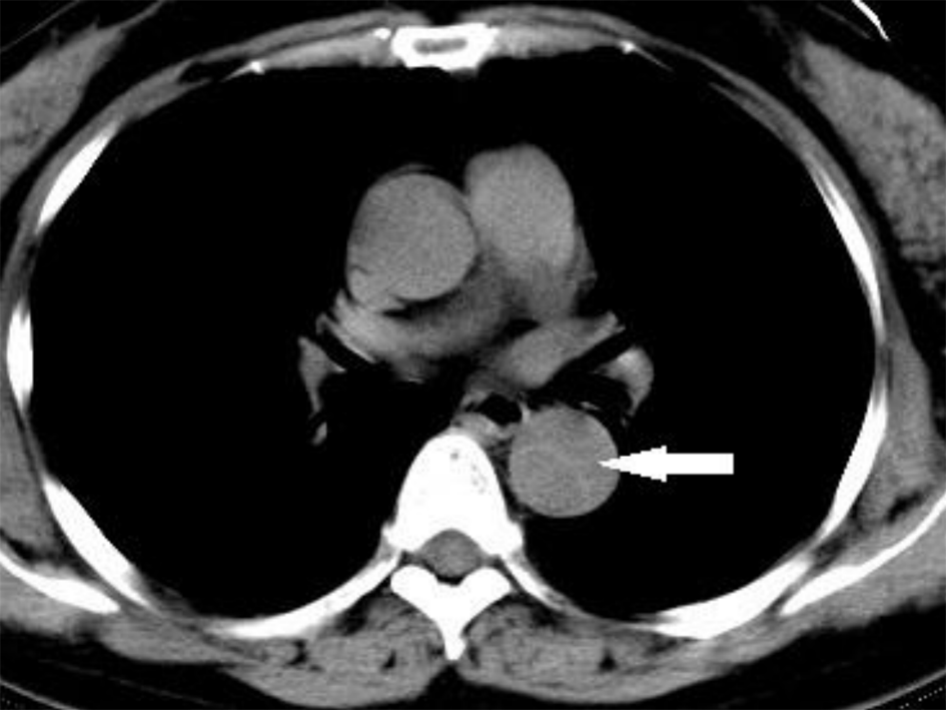
AAD found by chest CT; the arrow points to the inner wall of aortic blood vessel.

Patients with atypical symptoms in whom it was difficult to choose a diagnostic strategy underwent a CT or CTA for a rapid diagnosis. Rapid diagnosis of AAD is possible when CT or echocardiography are part of the diagnostic testing [4,6,17–19,21]. Thus, we performed imaging for patients with suspected AAD using a helical CT scanner of 64 or 16 to identify their diagnosis as soon as possible. In this study, CT was conducted in 32 patients (84.38% positive, all AAD patients diagnosed by CT in our hospital were confirmed by CTA in Nanjing Drum Tower Hospital) and CTA in 34 patients (94.12% positive). An example CT image is shown in Figure 3.

### 3.6. Outcome of AAD patients

Fifty-three patients with a diagnosis of AAD were transferred to Nanjing Drum Tower Hospital as soon as possible. Fifty-two of these patients survived, and there was 1 recorded death before arrival at Nanjing. Six patients were hospitalized in our hospital; 1 was treated with endovascular stent-graft placement, and 5 were administered drugs to control the disease.

## 4. Discussion

Failure to recognize the atypical symptoms of AAD may be one reason why patients without the manifestation of hypertension and severe chest pain experience longer delays in diagnosis than those with hypertension and typical chest pain. This could explain why AAD is reported to be the primary cause of death in these patients [1,2,7,9]. Timely diagnosis is essential for successful management; determination of the most important factors contributing to diagnostic and treatment delays is likely to improve the diagnostic and decision-making process. Around 15%–43% of patients later found to have AAD are initially suspected of having other conditions, such as acute coronary syndromes, nondissecting aneurysms, pericarditis, pulmonary embolism, or even cholecystitis [7,22]; thus, the differential diagnosis of AAD should be considered in patients presenting with atypical symptoms. We report that 37.29% of AAD patients are atypical, which is higher than in previous reports [23,24]. Furthermore, we found 25.42% of atypical patients to be without any pain, which is also higher than in some recent reports (6.4%–17%) [23,24]. A possible explanation is that atypical patients without pain in this study were at an earlier disease stage than those in other studies [23–25], which perhaps is attributed to our repeated education in the recognition of atypical AAD for our front-line physicians. The mean age of atypical patients in this study was 56.73 years, which was somewhat younger than in previous reports [23,24] and the Stanford classification of aortic dissection was not different between the two groups (P = 0.774); the reason for these is not clear.

So, how do we determine suspected AAD? When patients present with atypical symptoms increasingly frequently, or symptoms become aggravated despite symptomatic treatment, new atypical symptoms emerge during their stay in the emergency department. It becomes difficult to exclude the possibility of severe diseases; therefore, we pay more attention to certain high-risk illnesses such as AAD, pulmonary embolism, and acute coronary syndromes.

A combination of high D-dimer level [10–16] and atypical symptoms would be an effective method of diagnosing suspected AAD. A high level of LDH suggests hemolysis within a false lumen [1]; therefore, measurements of high levels of D-dimer, LDH, and CK were recommended in the initial management of patients with suspected AAD by the European Society of Cardiology [26]. CT or CTA was then used alongside a high measurement of D-dimer, LDH, and CK to confirm diagnosis of AAD. Before July 2015, we diagnosed 12 atypical AAD patients and found no statistical significance between atypical and typical groups, irrespective of the underlying mechanism of AAD, which is consistent with previous reports [1,26]. D-dimer levels below 500 µg/L may rule out suspected AAD if determined within the first 24 h after symptom onset [11,13]. A D-dimer level of <100 µg/L will exclude AAD in all cases (the lowest measurement in the present study was 160 µg/L, which is consistent with previous reports) [27]. The use of the D-dimer test to discriminate between AAD and acute myocardial infarction was effective in the emergency setting [28]; thus, we performed a D-dimer test for patients with suspected AAD without examination of LDH and CK from July 2015, which achieved satisfying results.

The atypical AAD symptoms found in this study were palpitations, tachypnea, obscure dread, light back and abdominal pain, unusual fatigue, feeling of impending death, and dyspnea. Palpitations, tachypnea, obscure dread, and light back and abdominal pain were usually the first atypical symptoms, followed by unusual fatigue, feeling of impending death, and dyspnea as secondary symptoms. Acute chest pain was absent in atypical AAD. Unusual fatigue and light back and abdominal pain were the most negligible and mild symptoms in these patients and were hardly associated with AAD. Tachypnea, obscure dread, feeling of impending death, and dyspnea were the severest of the symptoms; however, other diseases were often considered in patients with these symptoms before we studied their relation to AAD in-depth and provided enough education to our front-line physicians. The symptoms were often present for more than 1–2 days, sometimes lasting up to a week, resulting in the patient visiting the emergency department or clinic on repeated occasions. Laboratory and physical examinations often did not appear normal and symptomatic treatment was ineffective [24,29,30]. In this study, we describe the primary atypical symptoms of AAD as dyspnea, obscure dread, unusual fatigue, feeling of impending death, and light back and abdominal pain; palpitations and tachypnea are present in both the atypical and typical AAD groups. Previous studies have reported that dyspnea was a possible “clinical confounder” in making a diagnosis of painless AAD [24,29,30]. Palpitations as a symptom were previously described in a case report [29]; the other frequent symptoms and signs, including syncope, hemiplegia, pleural effusion, and pulse deficits, have also been previously described [24,29–31]. We did not observe these symptoms in our study, which may be because we identified these patients at an earlier stage [24,29,30].

The median delay duration for atypical AAD patients in our emergency department was 3.1 h (minimum: 1.0 h; maximum: 88.3 h; IQR: 5.4 h). The delay time in this study is shorter than in previous reports [9,23]. A definite diagnosis of AAD was made within 1 h of admission in only 4.55% of patients in the atypical group (Table 1); however, this trend has gradually changed as front-line physicians are increasingly aware to suspect AAD based on symptoms, physical findings, and results of initial diagnostic tests such as the D-dimer, LDH, and CK tests. The indications for clinical suspicion of AAD had been concluded by July 2015. After July 2015, the delay time (1.9 h) has improved greatly and was shorter than in some previous reports (9,23,24,30). This provides evidence that our method is effective in the diagnosis of atypical AAD, without putting patients at risk for misdiagnosis; however, the improved delay time (Table 1) did not reach statistical significance (P = 0.08). Furthermore, the essential education of front-line physicians in the recognition of the atypical symptoms of AAD may be helpful in the clinical decision of diagnosis.

Why do we choose CT or CTA as the first choice for AAD diagnosis [4,6,21]? Not only is it one of the most convenient examinations in our hospital and is accepted by common people in China, but it is also supported by the literature to help in the diagnosis of patients highly suspected of having AAD [7,9,32]. The improvement in diagnostic CT equipment may result in an increase in the number of cases accurately diagnosed with AAD [2,4–6]; thus we choose CT as a final examination in suspected AAD. In addition, our 16 helical CT scans are prepared for emergency patients 24 h a day, so more typical AAD patients were diagnosed in 2014 and 2015. The expense of CT is reasonable, and it is now the routine procedure to identify atypical and typical AAD in our emergency department.

Although we consider our findings to be valid, our study has several potential shortcomings. First, the major limitation of our study lies in the sample size; we showed that hospital-based databases, which include only patients who reach our hospital alive, would miss a substantial proportion of patients who failed to be delivered to hospital with AAD [3,9]. Second, some probable AAD patients with chest pain were considered to have myocardial ischemia and were subsequently hospitalized in the cardiac ward. Third, our echocardiography team is limited by their equipment, skills, and shifts, so this test was hardly used in this study. Fourth, the results of this data cannot be generalized to clinical sites that perform a dedicated accelerated diagnostic protocol as standard evaluation. It is likely that more atypical symptoms will be found in the future.

In conclusion, for patients with suspected atypical AAD, an evaluation of the atypical symptoms incorporated with a high level of D-dimer is used to decide on the execution of a prompt CT or CTA to diagnose these atypical patients, which could improve the efficiency of clinical decision making for the triage in the emergency department as compared with a standard evaluation strategy. Furthermore, the duration of the delay in the emergency department may be shortened. This method for atypical patients can be accomplished safely, without putting them at a greater risk for undetected AAD. Our study could allow physicians and patients to make informed decisions about the use of this strategy as an option for evaluation when symptoms are suggestive of AAD. The essential education directed towards the recognition of atypical symptoms of AAD for front-line physicians may be beneficial in clinical practice.

## Acknowledgment

We thank Jie Min, statistics teacher at the Medical College, Southeast University, Nanjing, Jiangsu, 210009, China, for her help with all the statistical calculations in this paper. This study was supported by Maanshan Technology Bureau (2016-RK02).
